# Physiological and Psychophysical Responses to Listening to Music during Warm-Up and Circuit-Type Resistance Exercise in Strength Trained Men

**DOI:** 10.1155/2015/389831

**Published:** 2015-08-04

**Authors:** Hamid Arazi, Abbas Asadi, Morteza Purabed

**Affiliations:** ^1^Department of Exercise Physiology, Faculty of Physical Education and Sport Sciences, University of Guilan, Rasht, Iran; ^2^Department of Physical Education and Sport Sciences, Payame Noor University, P.O. Box 19395-3697, Tehran, Iran

## Abstract

The purpose of this study was to assess the effects of listening to music during warm-up and resistance exercise on physiological (heart rate and blood pressure) and psychophysical (rating of perceived exertion) responses in trained athletes. Twelve strength trained male participants performed warm-up and resistance exercise without music (WU+RE without M), warm-up and resistance exercise with music (WU+RE with M), WU with M and RE without M, and WU without M and RE with M, with 48 hours space between sessions. After completing each session, the rating of perceived exertion (RPE) was measured. Also, heart rate (HR), systolic (SBP) and diastolic blood pressure (DBP), mean arterial pressure (MAP), and rate pressure product (RPP) were assessed before, after, and 15, 30, 45, and 60 min after exercise. Results indicated that RPE was higher for WU+RE without M condition in comparison with other conditions. All conditions showed increases in cardiovascular variables after exercise. The responses of HR, SBP, and RPP were higher for WU+RE without M condition. Thus, using music during warm-up and resistance exercise is a legal method for decreasing RPE and cardiovascular responses due to resistance exercise.

## 1. Introduction

Resistance training is important not only for athletes but also for members of the general population [1]. Many people use the weight training to improve their performance. Maximizing effort in the weight room can improve performance. Due to this, the use of ergogenic aids has become common place in exercise programs and resistance training. Some ergogenic aids can increase the capacity for bodily performance by inhibiting symptoms of fatigue such as imagery, caffeine, and steroids [2].

Several researchers have focused on the ergogenic aid of music during warm-up, exercise, and cool-down to enhance performance [3–6]. Music is considered to have a positive effect on exercise performance. Some have chosen to use music as an aid to physiological performance. Listening to music during resistance exercise is the good kind of distraction and can induce ups in the athletes effort, put the athletes in the peak effort, and elevate mood state resulting in greater work in resistance exercise movements. On the other hand, music has the capacity to capture attention, lift spirits, generate emotion, change or regulate mood, evoke memories, increase work output, reduce inhibitions, and encourage rhythmic movement all of which have potential applications in sport and exercise [2]. The most common positive outcomes when combining music and resistance exercise appear to be a decreased rating of perceived exertion, increased performance measures, improved mood, and increased arousal resulting in higher ability of weight lifts during resistance training and consequently better physiological and performance gains [4–8].

Another effect of listening to music is physiological (heart rate and systolic and diastolic blood pressure). Edworthy and Waring [9] found a significant increase in heart rate (HR) while listening to fast music during exercise. Conversely, Schwartz et al. [10] did not find any difference in exercise HR. Birnbaum et al. [11] reported that listening to fast music decreased the subjects' HR and blood pressure during steady-state treadmill exercise. In contrast, Jarraya et al. [12] investigated the effect of listening to music during warm up on short term supramaximal performance during the 30 s Wingate tests in athletes and found no significant changes in HR. Recently, Atan [3] examined effects of fast music tempo on HR and found no significant changes. The contradictory findings may be due in part to variations in experimental design, such as differences in exercise mode, intensity, and duration. The type of music selected and the manner in which it was selected have also varied among the studies. Additionally, subjects differed in age, health, and physical conditioning.

It is widely known that the autonomic nervous system is involved in control of HR and cardiovascular responses. That is, increased sympathetic neural activity plays a role in controlling the rate and contractility of the heart and in the caliber of resistance and capacitance vessels in the systemic circulation [13]. Music also appears to affect the autonomic nervous system [14]. Therefore, physiological reactions should result from changes in the autonomic nervous system triggered by music.

Although previous studies supported the hypothesis of the ergogenic effects of music on cardiovascular performance, it is important to examine music's effect with trained athletes and using music during warm-up and circuit-type resistance exercise. The effects of music played during an exercise task on athletic performance have been previously studied. Yet, these results are not applicable for well-trained athletes and using music during warm-up and circuit-type resistance exercise with emphasis on physiological aspects. Therefore, the aim of this study was to compare four conditions of combining music and resistance exercise on physiological and psychophysical responses in trained athletes.

## 2. Material and Methods

### 2.1. Participants

Twelve healthy well-trained resistance exercise males (Table 1) volunteered to participate in this investigation. None of the athletes were on any medication. All participants were free from any lower and upper body injuries and cardiovascular diseases and did not use anabolic or other drugs that enhance the performance for at least 6 months prior to involvement in this study. Inclusion criteria included (a) at least 5 years of experience with resistance training, (b) no history of lower and upper body injuries, (c) no lower and upper extremity reconstructive surgery in the past two years or unresolved musculoskeletal disorders, and (d) not using any ergogenic aids throughout the study. Before any measurements and starting the study, each athlete gave written informed consent to participate. All participants understood that they could withdraw from the study at any time. This study was approved by the Local Ethics Committee and procedures were conducted in accordance with ethical standards in sport and exercise science research [15].

### 2.2. Experimental Design

At least one week prior to starting the treatments, all athletes reported to the laboratory, and during this session their body weight and height were determined in a standardized fashion. Height was measured in centimeters using a stadiometer (Seca 222, Terre Haute, IN) and body mass was measured in kilograms using a digital scale (Tanita, BC-418MA, Tokyo, Japan). At this session, the participants understood the procedures and experimental approach of this study and performed selected resistance exercises for familiarization, and then the one repetition maximum for each exercise was measured following NSCA guidelines [16]. After the first session, the athletes reported to laboratory four times and performed each condition (treatment). Each exercise session took place at approximately the same time of day for each athlete and sessions were separated by at least 48 hours.

### 2.3. Procedures

The research design consisted of four experimental conditions. Each athlete was tested four times under the same laboratory conditions in a randomized manner. Condition 1 (WU+RE without M) consisted of performing warm-up and resistance exercise without music. For condition 2 (WU+RE with M) the athletes listened to music during warm-up and while performing resistance exercise. Condition 3 (WU with M+RE without M) consisted of performing warm-up with M and performing resistance exercise without M, and for condition 4 (WU without M+RE with M), the athletes performed warm-up without listening to music and listened to music during resistance exercise.

Total time of warm-up was 10 minutes including five minutes of cycling on a cycle-ergometer (Sport Art Fitness, C52u, Taiwan) at a self-selected workload and cadence and five minutes of various stretching exercises with the aim of preparing the joints for resistance exercises. Resistance exercises included the squat, military press, leg press, lat pull dawn, knee flexion, bench press, knee extension, biceps extension, calf raise, and arm curl. The athletes performed a set of 10 repetitions with 60% of one repetition maximum, continually and without rest between stations. Only one circuit was performed by each participant.

Due to the exercise type of resistance exercise and the relationship between arousal and sympathetic nervous system with resistance exercise, fast (130 beats per minute [bpm]) tempo music was chosen for this study. The selection criteria were based on the five recommendations of Karageorghis and Terry [17]. The motivational effects of asynchronous music and an associated measure of the motivational qualities of music known as the Brunel Music Rating Inventory (BMRI), with regard to the authors who indicated the main characteristics of motivational music are that it has a fast tempo (>120 bpm) and strong rhythm, which increase energy and induce bodily action (3, 18, 22). Music was played from five speakers connected to a computer. For conditions 2, 3, and 4, the music was played during the specified period. For example, in the WU without M+RE with M (condition 4) warm-up was performed without any music and when the athletes performed the resistance exercise, the music was played until the participants completed the circuit.

The RPE was assessed using CR-15 RPE scale. The RPE scale has been shown to be a valid instrument in which to evaluate perceived exertion and quantification of resistance exercise intensity in a variety of populations. Citation numbers from 6 to 20 on the scale were used to rate the intensity of the entire workout session. A rating of 6 was associated with rest, and the highest rating, 20, referred to maximal effort. After completing a circuit of resistance exercises, the athlete was asked “How would you rate your effort?” The participants would verbally indicate a number to rate their overall effort based on score of 6 to 20 [18].

Heart rate and blood pressure were measured before starting the conditions in a seated position in a quiet and comfortable place (prevalue). After performing each condition, the athletes' heart rate and blood pressure were measured every 15 minutes for 60 minutes. During postexercise assessment, the participants maintained seated positions in a quiet and comfortable place. Heart rate (HR) was measured using a Polar S610i HR monitor (FIN, 90440, Finland).

Blood pressure was measured by the same experienced observer using a standard mercury sphygmomanometer (Missouri) and stethoscope (Rappaport GF Health Products, Northeast Parkway Atlanta). Systolic blood pressure (SBP) was determined as the appearance of Korotkoff sounds, while the point of disappearance of these sounds was considered to be the diastolic blood pressure (DBP). Mean arterial pressure (MAP) was calculated as DBP + [0.333 (SBP − DBP)]. The rate pressure product (RPP) was calculated as SBP × heart rate. It is considered a reliable predictor of myocardial oxygen demand [19].

### 2.4. Statistical Analysis

Data are presented as means ± SD. Prior to analysis, data normality was checked with the Kalmogorov-Smirnoff test. Repeated measures analysis of variance (ANOVA) was used for determining differences. When a significant *F* value was achieved, the Bonfferoni post hoc test was used to analyze the RPE, HR, SBP, DBP, MAP, and RPP responses after different conditions to determine where the difference occurred. The level of significance was set at *p* < 0.05 for all statistical procedures. All analyses were conducted using SPSS version 16.0 (SPSS Inc., Chicago, IL, USA).

## 3. Results

**Figure 1:**
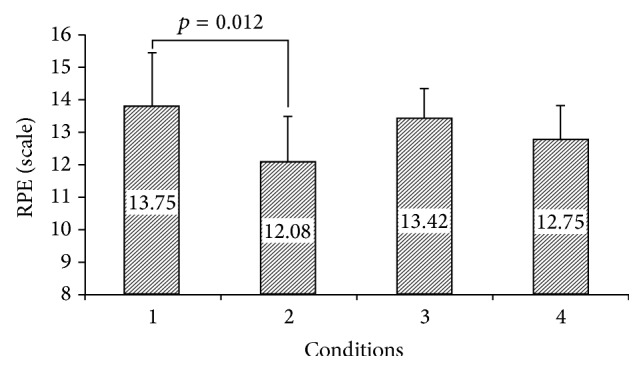
Rating of perceived exertion following 4 conditions; 1: WU+RE without M, 2: WU+RE with M, 3: WU with M+RE without M, and 4: WU without M+RE with M.

The repeated measures ANOVA showed a significant difference among the mean RPE values of the conditions (*F*_2.02,22.2_ = 7.25, *p* = 0.004) as condition 1 displayed an increase in perceived exertion greater than condition 2. RPE values are displayed in Figure 1.

**Figure 2:**
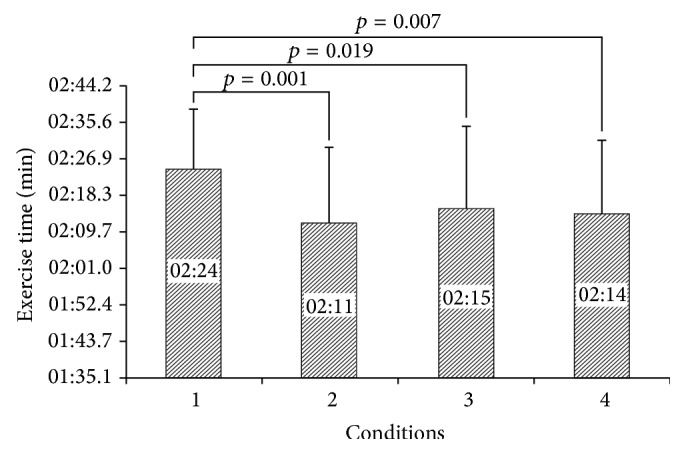
Completion time for a circuit resistance exercise (mean ± SD). 1: WU+RE without M, 2: WU+RE with M, 3: WU with M+RE without M, and 4: WU without M+RE with M.

Total time of completing a circuit of resistance exercise was higher for condition 1 compared to the other conditions (*F*_2.02,22.88_ = 16.63, *p* = 0.001) (Figure 2).

Heart rate, SBP, DBP, MAP, and RPP values for each condition are presented in Table 2. For HR, the main effect of resistance exercise was significant increases in HR postexercise and 15 minutes after exercise in all conditions. Likewise condition 1, condition 2, and condition 3 displayed these increases in HR until 30 minutes after circuit-type resistance exercise (*p* = 0.001). Only condition 1 showed incremental increases in HR until 45 minutes after exercise when compared to preexercise values (*p* = 0.017). There were no significant differences among conditions in HR at any time point after exercise (*F*_5.43,79.72_ = 0.698, *p* = 0.639).

No significant differences were observed at any time points of postexercise SBP (*F*_9.97,146.26_ = 0.943, *p* = 0.496), DBP (*F*_9.70,142.4_ = 0.366, *p* = 0.957), MAP (*F*_5.02,73.76_ = 0.741, *p* = 0.596), and RPP (*F*_3.17,46.58_ = 0.982, *p* = 0.413) when the conditions were compared.

SBP and MAP increased significantly after exercise in comparison to before exercise (*p* = 0.001) for all conditions. Likewise, these increases for condition 1 (*p* = 0.026, *p* = 0.023) and condition 2 (*p* = 0.018, *p* = 0.037) continued until 15 minutes after exercise, respectively. Condition 1 showed incremental increases in SBP until 30 minutes after exercise when compared to preexercise values (*p* = 0.015).

The changes in DBP at all time points were not statistically significant for all conditions. For RPP, all conditions showed significant increases until 30 minutes after exercise when compared to preexercise values (*p* = 0.001), and only condition 1 maintained their increased RPP until 45 minutes after circuit-type resistance exercise (*p* = 0.001).

## 4. Discussion 

The aim of this study was to examine the effects of listening to music on warm-up and circuit-type resistance exercise. The results showed that the rating of perceived exertion was higher for participants who performed both warm-up and resistance exercise without music in comparison with other conditions that played music for at least the warm-up or resistance exercise or both. There were significant differences between WU+RE without M and WU+RE with music in perceived exertion that support music as ergogenic aid. The results of the present study are consistent with those of previous studies that reported reduced RPE while listening to music. Nethery et al. [20], Potteiger et al. [7], Nethery [8], Yamashita et al. [4], and Dyer and McKune [5] reported a decrease in RPE when listening to music while exercising. In contrast, Schwartz et al. [10], Caria et al. [21], Birnbaum et al. [11], and Chtourou et al. [22] did not find any significant differences in RPE when their subjects listened to music. These differences in findings could be at least partly attributed to differences in exercise intensity, duration, trained and untrained states of the participants, and the music selection process. Overall, with regard to the positive effects of listening to music in decreasing RPE in this study, enhancing arousal levels and facilitating motor coordination could be possible mechanisms for this effect. However, we did not measure these features specifically but previous studies reported this [4–8, 20]. With regard to significant differences between WU+RE without M and other conditions in completing a circuit resistance exercise time, it seems that increasing arousal during listening to music played a critical role in completing the resistance exercise session in minimal time. However, some authors reported no significant effects of music on completion time of exercise [5]. Consequently, further studies on the effect of music on exercise completion time are necessary.

To date, this study was the first investigation that examined the effects of music during warm-up or circuit-type resistance exercise on physiological or cardiovascular responses (HR, SBP, DBP, MAP, and RPP) in well-trained athletes. The results showed that postexercise HR, SBP, MAP, and RPP significantly increased in comparison with before exercise for all conditions. Likewise, RPP and HR were high until 15 minutes after exercise in all conditions. Moreover, HR, SBP, and RPP remained elevated until 30-to-45 min after exercise for the WU+RE without M condition. These findings revealed the ergogenic effect of music for decreasing cardiovascular responses to exercise. This is a surprising finding because of the common notion that music serves as a distraction from the exercise itself, and other investigators have reported an increase in HR and blood pressure due to stimulation of the sympathetic nervous system when listening to music while exercising [4, 11, 13, 14]. Edworthy and Waring [9] and Thornby et al. [23] observed a significant increase in HR when their subjects listened to fast music while exercising. Their studies used different exercise protocols and different music selection processes, which may account for the contradictory findings. The discrepancy between our findings and previous studies could be differences in exercise type (circuit-type resistance exercise versus treadmill or cycle), duration, training status of the subjects, and the music selection process (fast versus medium or low tempo).

The limitation of the study was the number of subjects. The limitations of this study include the low number of subjects which prevents generalization of the results. Additionally the results of the current investigation are based on nonsporting population, and further research is needed to determine if similar effects are obtained in the sporting population and during sport events. Moreover, there is no financial support for this project and we did not measure some of blood indicators, which can be a subject for further researches. In this study we used only one circuit and for further studies the researchers can increase number of circuits (i.e., 3 times).

## 5. Conclusions

In light of these study findings, fast tempo music (130 bpm) may decrease perceived exertion in well-trained individuals during a warm-up when completing circuit resistance exercise training. Likewise, music may be considered as a legal ergogenic aid for well-trained athletes during warm-up prior to resistance exercise and during resistance exercise for decreasing perceived exertion and minimizing training time. Thus, professional strength and conditioning coaches and practitioners interested in resistance exercise may be well advised to incorporate arousing music during warm-up and exercise sessions. Music may decrease physiological and/or cardiovascular responses in athletes while performing resistance exercise. Therefore, it can be recommend that coaches and strength and conditioning professionals use music during warm-up and resistance exercise for the decreases of RPE and cardiovascular responses.

## Figures and Tables

**Table 1:** Participants characteristics and resting cardiovascular parameters (mean ± SD).

Number of athletes	12
Age (y)	24 ± 2
Height (cm)	175.1 ± 6.4
Weight (kg)	83.7 ± 11.2
Strength training experience (y)	5.2 ± 1.3
Heart rate (bpm)	70.6 ± 6.8
Systolic blood pressure (mmHg)	120.1 ± 7.3
Diastolic blood pressure (mmHg)	79.7 ± 3.1

**Table 2:** Heart rate (HR), systolic blood pressure (SBP), diastolic blood pressure (DBP), mean arterial pressure (MAP), and rate pressure product (RPE) responses to warm-up and resistance exercise with and without of music. Values are mean ± SD.

Conditions		Variable/time				
		HR (bpm)				
	Before	After^*∗*^	15 min^*∗*^	30 min^†^	45 min^§^	60 min

Condition 1	69.8 ± 7.7	133.9 ± 18.1	96.1 ± 9.8	80.5 ± 8.7	78.5 ± 9.3	75 ± 9.9
Condition 2	72.6 ± 10.5	138.8 ± 16.4	95.6 ± 9	78.5 ± 10.1	73.4 ± 8.6	69.5 ± 8.2
Condition 3	68 ± 8.4	131.5 ± 22.6	93.8 ± 14.6	79.3 ± 12	72.6 ± 10.6	69.5 ± 8.9
Condition 4	72 ± 7.1	134.3 ± 16.6	93.6 ± 11.8	77.8 ± 10.8	72.1 ± 8.4	70.6 ± 9.3

		SBP (mmHg)				
	Before	After^*∗*^	15 min^‡^	30 min^§^	45 min	60 min

Condition 1	116.9 ± 6.7	142 ± 8.3	129.1 ± 10.1	124.4 ± 9.9	120.8 ± 7.3	115.9 ± 6.2
Condition 2	116.1 ± 6.7	141.9 ± 9.4	125.8 ± 9.9	120.1 ± 8	117.7 ± 6.8	115.8 ± 5.5
Condition 3	120.7 ± 8.1	146 ± 7.5	129.3 ± 7.7	126.4 ± 7.2	119.3 ± 6.3	119.2 ± 6.5
Condition 4	120.7 ± 8.1	149.5 ± 6.2	131.2 ± 9.5	122.9 ± 7.8	119.8 ± 6.8	119.5 ± 7.5

		DBP (mmHg)				
	Pre	Post	15-min	30-min	45-min	60-min

Condition 1	78.7 ± 3.1	84.7 ± 4.3	80.9 ± 2.1	81.2 ± 5.2	79.1 ± 3.2	79.5 ± 2.5
Condition 2	80 ± 2.1	85.4 ± 4.5	82.5 ± 3.9	80.2 ± 2.6	80 ± 2.1	79.5 ± 1.4
Condition 3	79.9 ± 1.7	85 ± 3.6	81.8 ± 3.2	81.6 ± 4.9	80 ± 2.1	79.7 ± 1.6
Condition 4	80.5 ± 3.9	84.8 ± 5.4	81.8 ± 3.2	82.2 ± 3.2	80.5 ± 1.5	80 ± 1

		MAP (mmHg)				
	Before	After^*∗*^	15 min^‡^	30 min	45 min	60 min

Condition 1	97.8 ± 3.9	113.4 ± 5.6	104.8 ± 5.6	102.6 ± 6.2	92.4 ± 6.5	97.7 ± 3.5
Condition 2	98 ± 3.5	113.6 ± 5.9	104.1 ± 6.2	100.2 ± 4.4	99.9 ± 3.8	99.5 ± 3.8
Condition 3	100.3 ± 4.4	115.5 ± 4.4	105.5 ± 4.5	104.4 ± 5.2	99.6 ± 3.4	99.5 ± 3.3
Condition 4	100.6 ± 5.1	116.1 ± 3.6	106.5 ± 5.5	102.5 ± 4.4	100.2 ± 3.1	99.7 ± 3.7

		RPP (bpm × mmHg)				
	Before	After^*∗*^	15 min^*∗*^	30 min^*∗*^	45 min^‡^	60 min

Condition 1	8190 ± 1224.4	19118 ± 3518.9	12434 ± 1784.4	10067 ± 1684.4	9512 ± 1422.7	8735 ± 1429
Condition 2	8449 ± 1348.6	19790 ± 3377.6	12077 ± 1854	9471 ± 1557	8665 ± 1286.9	8073 ± 1122
Condition 3	8245 ± 1319	19222 ± 3512.9	12567 ± 1741.2	10047 ± 1711.1	8698 ± 1523.4	8320 ± 1394.5
Condition 4	8707 ± 1175.5	19789 ± 2436.1	12305 ± 1878	9591 ± 1612.7	8674 ± 1313.5	8498 ± 1552.2

Condition 1: WU+RE without M, condition 2: WU+RE with M, condition 3: WU with M+RE without M, and condition 4: WU without M+RE with M. ^*∗*^Significant differences in comparison with prevalue for all conditions; ^†^significant differences in comparison with prevalue for conditions 1, 2, and 3; ^‡^significant differences in comparison with prevalue for conditions 1 and 2; ^§^significant differences in comparison with prevalue for condition 2.
